# Staged strategy of combined rotational atherectomy and intravascular lithotripsy for severely calcified lesions: an evaluation using multimodality intracoronary imaging—a case report

**DOI:** 10.1093/ehjcr/ytae504

**Published:** 2024-09-19

**Authors:** Yusuke Miura, Kohei Koyama, Keiichi Izumi, Hiroyuki Yamazaki, Kyoko Soejima

**Affiliations:** Department of Cardiovascular Medicine, Kyorin University Hospital, 6-20-2 Shinkawa, Mitaka, Tokyo 181-0004, Japan; Department of Cardiovascular Medicine, Kyorin University Hospital, 6-20-2 Shinkawa, Mitaka, Tokyo 181-0004, Japan; Department of Cardiovascular Medicine, Kyorin University Hospital, 6-20-2 Shinkawa, Mitaka, Tokyo 181-0004, Japan; Department of Cardiovascular Medicine, Kyorin University Hospital, 6-20-2 Shinkawa, Mitaka, Tokyo 181-0004, Japan; Department of Cardiovascular Medicine, Kyorin University Hospital, 6-20-2 Shinkawa, Mitaka, Tokyo 181-0004, Japan

**Keywords:** Case report, Intravascular lithotripsy, Rotational atherectomy, Calcified lesions, Near-infrared spectroscopy

## Abstract

**Background:**

Severely calcified lesions are the most significant challenge for percutaneous coronary intervention, exhibiting poor clinical outcomes. Some severely calcified lesions remain untreatable with conventional balloons or even atherectomy devices. Intravascular lithotripsy is a new option for treating severe calcification.

**Case summary:**

Herein, we describe a case of ischaemic cardiomyopathy with a thick, circumferential calcified lesion in the proximal and mid-segments of the left anterior descending coronary artery. In the first session, high-pressure balloons, cutting balloons, and rotational atherectomy failed to disrupt the calcification. In the staged additional treatment that was subsequently planned, eight cycles of intravascular lithotripsy created multiple fractures in the deep calcification, resulting in successful stent deployment. The effect of intravascular lithotripsy was observed mainly in calcified areas with lipid components detected using near-infrared spectroscopy-intravascular ultrasound.

**Discussion:**

Our report suggests the efficacy of employing a combined strategy of rotational atherectomy with small burrs and intravascular lithotripsy in the treatment of severe calcification with a minimal risk of complications. Our study introduces a novel aspect by utilizing near-infrared spectroscopy-intravascular ultrasound to evaluate calcified lesions before performing intravascular lithotripsy. To our knowledge, there have been no similar reports to date. The effect of intravascular lithotripsy on calcified lesions may be related to the distribution of lipid components and/or heterogeneity within the calcification.

**Learning points**

- The combined strategy of rotational atherectomy with small burrs and intravascular lithotripsy in the treatment of severe calcification could yield procedural and clinical outcomes with a minimal risk of complications.
- Utilization of near-infrared spectroscopy-intravascular ultrasound enhances pre-procedural assessment, offering valuable insights into calcified lesions’ composition and aiding in strategic planning for interventions like intravascular lithotripsy.
- The observed relationship between lipid components within calcified lesions and the impact of intravascular lithotripsy suggests a potential correlation, emphasizing the need for further exploration to elucidate its mechanism and optimize treatment strategies.

## Introduction

Percutaneous coronary intervention (PCI) for severely calcified lesions does not produce satisfactory results regarding early success rate and chronic stent patency.^1,2^ Although rotational atherectomy (RA) has long been used to treat severely calcified lesions, its use in treating thick, deep, and circumferential calcified lesions is limited. Intravascular lithotripsy (IVL) is an entirely different approach, providing a new option for treating severe calcification.^3^ This report describes a case of a severely calcified lesion successfully treated with a staged strategy combining RA and IVL.

## Summary figure

**Figure:**
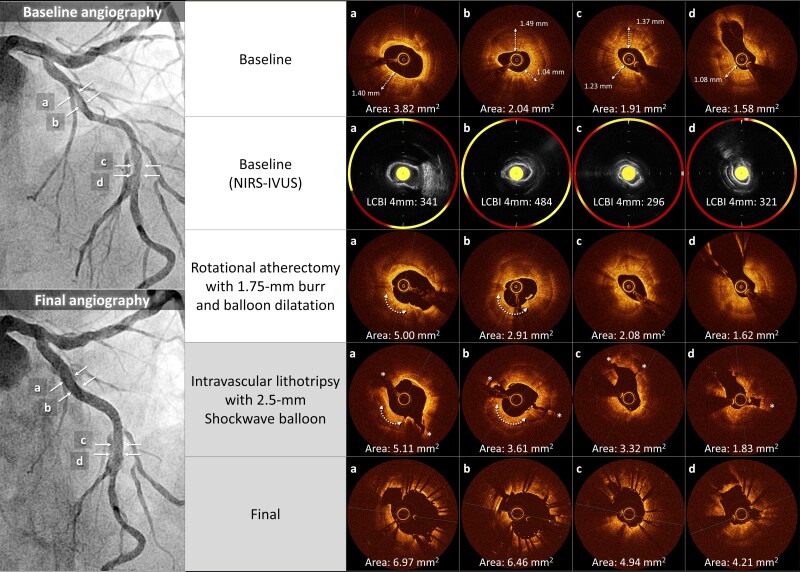


## Case report

**Figure 1:**
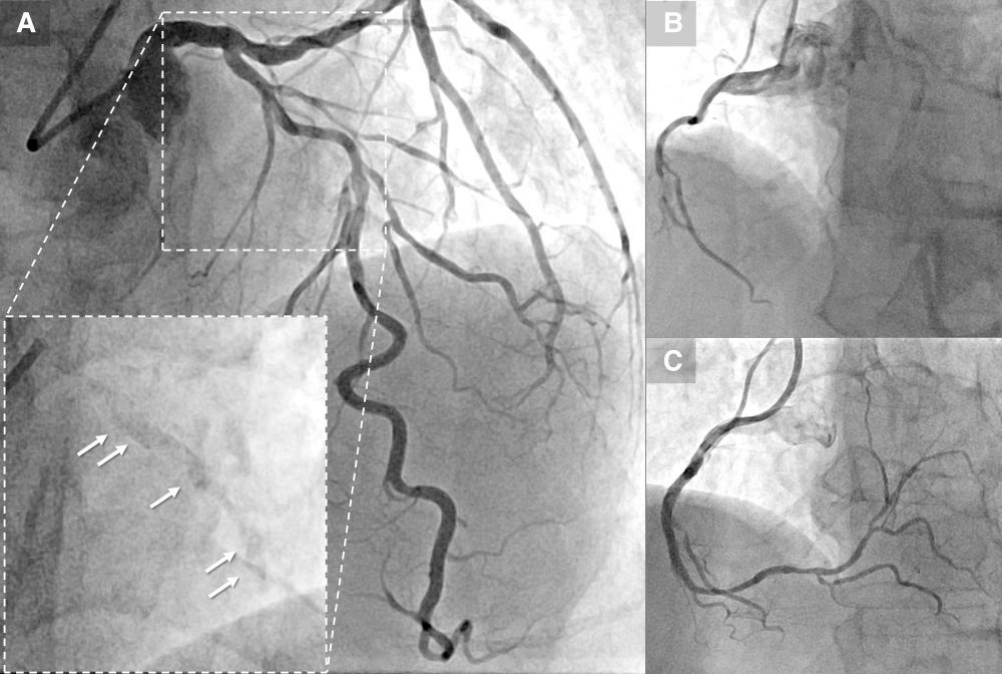
(*A*) Coronary angiography reveals stenosis with severe calcification in the proximal and mid-segment of the left anterior descending, clearly visible prior to contrast injection (arrow). (*B*) Sub-total occlusion in the mid-right coronary artery. (*C*) Post–percutaneous coronary intervention angiography of the right coronary artery. LAD, left anterior descending; PCI, percutaneous coronary intervention; RCA, right coronary artery.

**Figure 2:**
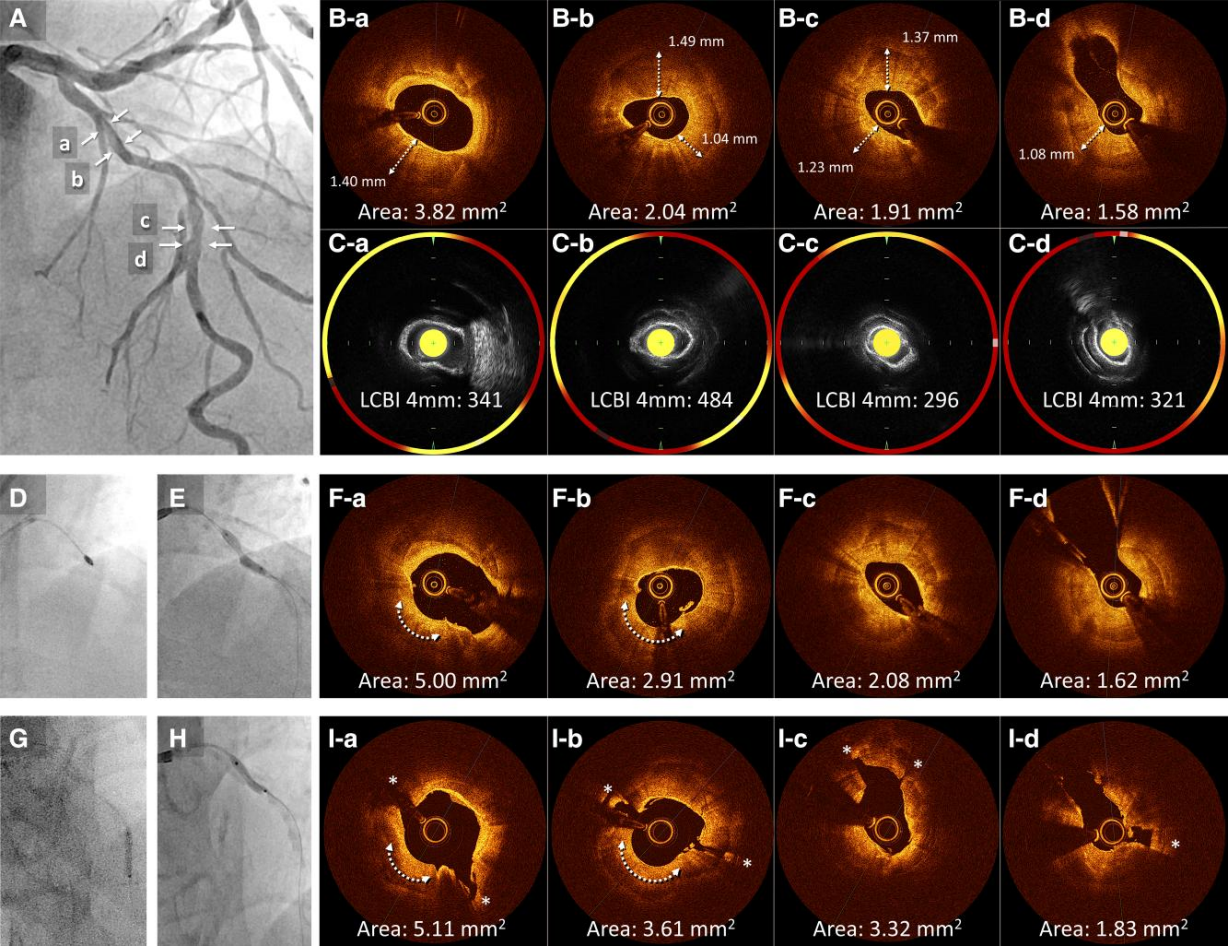
(*A*) Baseline angiography of the left anterior descending in the first session. (*B*) Baseline optical coherence tomography shows circumferential calcification with a thickness exceeding 1.0 mm in the proximal (a and b) and mid-lesions (c and d). (*C*) Near-infrared spectroscopy-intravascular ultrasound showing lipid components inside or behind the calcification (yellow areas). (*D*) Rotational atherectomy with 1.5 and 1.75 mm burrs is performed on the proximal lesion. (*E*) After Rotational atherectomy, the proximal lesion is still undilatable. (*F*) Optical coherence tomography after rotational atherectomy and balloon dilation reveals residual heavy calcification with limited lumen expansion and no calcium fractures, although the ablation effect by rotational atherectomy was observed at the 7 o’clock position of the proximal lesion (arrow). (*G* and *H*) Lithotripsy for eight cycles is performed sequentially from the mid- to the proximal left anterior descending. (*I*) Subsequent optical coherence tomography reveals multiple major disruptions (*). LAD, left anterior descending; NIRS-IVUS, near-infrared spectroscopy-intravascular ultrasound; OCT, optical coherence tomography; RA, rotational atherectomy.

**Figure 3:**
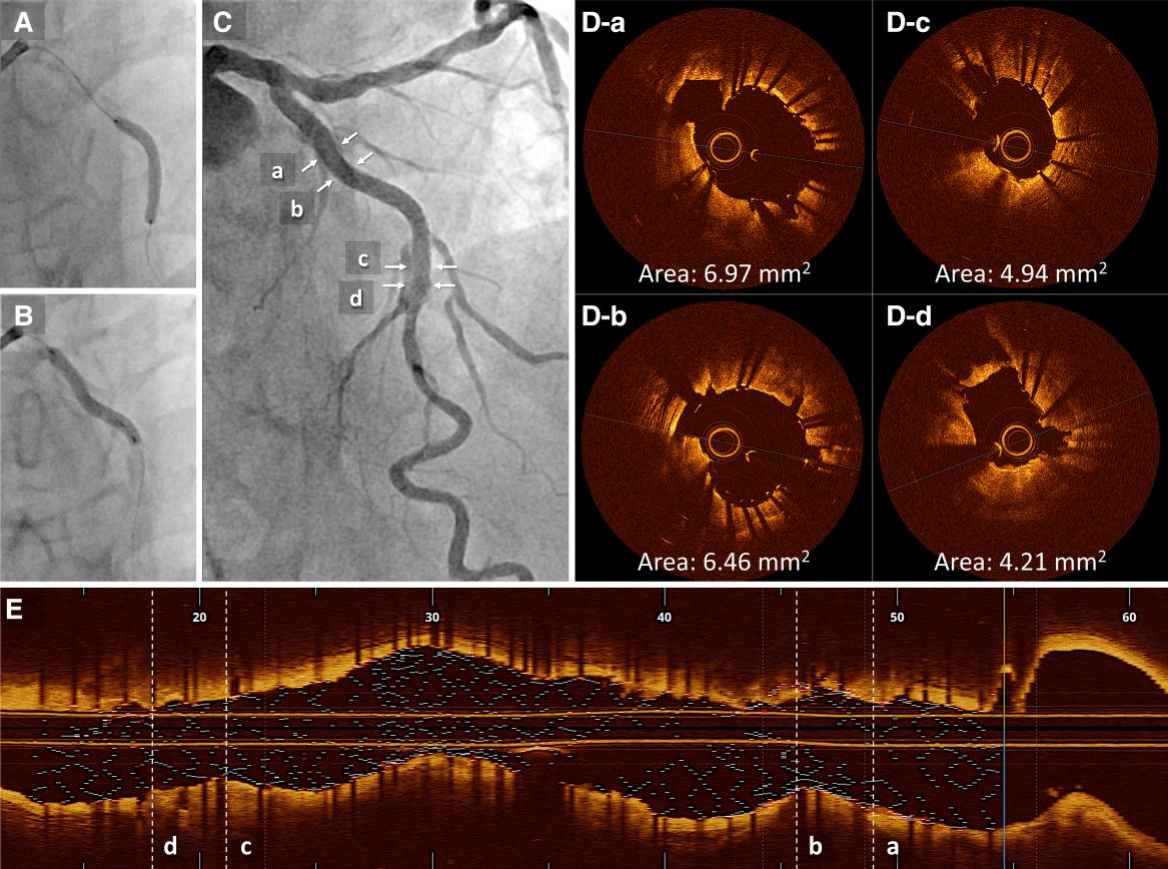
(*A* and *B*) Two drug-eluting stents (2.5 × 22 mm and 3.0 × 26 mm) are deployed overlapping. (*C*) The final angiography result is acceptable. (*D*) Post-procedural optical coherence tomography. (*E*) The final longitudinal reconstructed optical coherence tomography image. MSA, minimum stent area; OCT, optical coherence tomography.

A 76-year-old man with a history of hypertension and smoking presented to our outpatient clinic with symptoms of chest pain on exertion. Electrocardiography revealed atrial fibrillation with a complete left bundle branch block. Echocardiography demonstrated a left ventricular ejection fraction (LVEF) of 32% and impaired wall motion from the anteroseptal and inferior wall with no structural heart disease. Coronary angiography showed 75% stenosis with severe calcification in the proximal and mid-segment of the left anterior descending (LAD) artery (*Figure 1A*, Supplementary material online, *Video 1*) and a sub-total occlusion in the mid-right coronary artery (RCA) with collateral flow from the septal branches (*Figure 1B*). Optimal medical therapy was initiated, and PCI to the RCA was performed first (*Figure 1C*). Physiological assessment of the LAD artery showed positive ischaemia, with a resting full-cycle ratio of 0.82; thus, PCI to the LAD was subsequently performed (*Figure 2A*). Optical coherence tomography (OCT) revealed full circumferential calcification with a thickness exceeding 1.0 mm in the proximal and mid-LAD lesions, with minimum lumens of 2.04 mm^2^ and 1.58 mm^2^, respectively (*Figure 2B*). Near-infrared spectroscopy-intravascular ultrasound (NIRS-IVUS) was also performed to evaluate the lesions (*Figure 2C*). The NIRS-IVUS detected lipid components inside or behind the calcification. Rotational atherectomy of the proximal lesion was performed with 1.5 and 1.75 mm burrs (Boston Scientific, MA, USA), followed by balloon dilatation (*Figure 2D*). Despite great difficulty, a 3.0 mm high-pressure non-compliant (NC) balloon (24 atm) and a 3.0 mm Wolverine^TM^ Cutting Balloon^TM^ (Boston Scientific) were successfully delivered with the support of a guide extension catheter; however, the proximal LAD lesion was still undilatable (*Figure 2E*). Optical coherence tomography revealed the definite ablative effect of RA on superficial calcification, whereas the effect on deep calcification was minimal, resulting in failure to achieve effective calcium disruption with NC balloon dilatation (*Figure 2F*). Since stent deployment was considered inappropriate, the procedure was finished with dilatation of a 3.0 × 30 mm drug-coated balloon. Despite the up-titration of optimal medical therapy, the LVEF remained unimproved with residual angina, and the patient was hospitalized for worsening heart failure due to anaemia, with a haemoglobin level decreasing from 12.2 g/dL (baseline) to 6.4 g/dL. Although the aetiology of the anaemia was undetermined even with gastrointestinal endoscopy, colonoscopy, and bone marrow examination, heart failure was compensated with blood transfusions and diuretics. Additional PCI to the LAD was considered, but whether effective de-bulking was possible even with RA using burrs >2.0 mm was uncertain; the femoral artery approach, with its high bleeding risk, was also a concern. Orbital atherectomy (OA) carries a potential risk of coronary arterial injury related to tortuosity around the lesions. Therefore, a second PCI session for LAD was planned after IVL became available at our institution. Baseline angiography revealed no significant changes compared with the findings at the end of the first session. Via a trans-radial approach, the procedure was initiated using a 7 Fr SPB 3.5 guide catheter (ASAHI INTECC, Aichi, Japan) and a 0.014 in. SION blue ES guidewire (ASAHI INTECC). With the support of a guide extension catheter, a 2.5 mm Shockwave IVL balloon (Shockwave Medical, CA, USA) was delivered to the mid-LAD lesion, and eight cycles of lithotripsy were sequentially performed up to the proximal LAD (*Figure 2G* and *H*). Subsequent OCT revealed multiple fractures in the calcium lesions (*Figure 2I*, Supplementary material online, *Video 2*). In comparison with the NIRS-IVUS findings of the first session, major fractures were observed to occur mainly in the calcified areas with abundant lipid components. Consequently, the proximal lesion was successfully dilated with a 3.0 mm NC balloon. Finally, two drug-eluting stents (2.5 × 22 mm and 3.0 × 26 mm) were deployed, overlapped, and post-dilated with a 3.5 mm NC balloon proximally and a 3.0 mm NC balloon distally (*Figure 3A* and *B*). The procedure was completed with excellent angiographic results without complications (*Figure 3C*, Supplementary material online, *Video 3*). The final OCT revealed an acceptable result for both proximal and mid-LAD lesions, with a stent expansion rate of 82% (*Figure 3D* and *E*). The patient made good progress after 3 months with no angina or heart failure symptoms, with an improved LVEF of 53%.

## Discussion

The use of PCI for calcified lesions is rapidly increasing, accounting for ∼30% of all procedures.^4^ Severely calcified lesions remain the most significant challenge for PCI; they increase procedural complexity and risk, resulting in stent thrombosis, in-stent restenosis, and target lesion revascularization.^1,2^ Therefore, optimal lesion preparation is crucial for improved outcomes in treating calcified lesions.^5^ Conventional procedures for calcified lesions include high-pressure NC balloons, scoring balloons, cutting balloons, and atherectomy devices. In cases of circumferential calcification, balloon dilation alone is usually ineffective, especially for thick and deep calcifications. Rotational atherectomy and OA have significantly improved the effectiveness of treating severely calcified lesions. Still, these procedures require specialized expertise and have the potential risk of serious complications, including slow flow, perforation, and dissection.^6,7^ Furthermore, RA and OA primarily ablate superficial calcium eccentrically following the guidewire bias, thus limiting circumferential ablation and efficacy on deep calcifications. Hence, some calcified lesions remain unresolved even with these modalities.

Intravascular lithotripsy is a novel device that disrupts deep calcification with circumferentially emitted sound waves and low-pressure balloon dilation, providing a new option for treating severely calcified lesions.^3,8^ Since high-pressure dilation is not required, interaction with surrounding soft tissue is minimal, and the procedure can be performed more safely via a radial artery approach.^9^ Moreover, unlike RA and OA, IVL is unaffected by guidewire bias, allowing circumferential disruption of calcified plaques, and is also effective for deep calcifications. However, the IVL system has a more extensive entry and crossing profile than conventional balloons, making it difficult to deliver to tight calcified lesions.

In the present case, circumferential deep calcification of the proximal LAD had a maximum thickness of 1.49 mm and a length of 17.2 mm, with a calcium score of 4.^10^ Multiple RAs with 1.5 and 1.75 mm burrs aided the deliverability of large NC balloons but could not fracture the thick calcification. As a result, stent deployment was unsuccessful. In the second session, eight cycles of IVL provided multiple calcification fractures, allowing for stent deployment. The differential effect of RA and IVL on calcified lesions was observed on OCT images. Rotational atherectomy had a volume reduction effect on calcification following the wire bias, whereas IVL showed a disruptive effect irrespective of calcification thickness. Rotational atherectomy was consequently effective in delivering the IVL balloons in the first session.

The novelty of the presently reported case is the use of NIRS-IVUS to evaluate calcified lesions in the first session, and to our knowledge, there have been no similar reports to date. Since near-infrared light penetrates calcium, it can detect lipid components inside or behind calcifications, which is difficult to detect using conventional IVUS or OCT.^11^ The calcification-disrupting effect of IVL in the second session was mainly observed in areas where the presence of lipid components was suggested by NIRS-IVUS in the first session. It is possible that the internal structure of the calcification may influence IVL’s disruptive effect. Some types of calcified lesions have been pathologically demonstrated to have lipid pools or necrotic cores inside.^12^ If the effectiveness of IVL varies between calcifications with softer internal components compared to fibro-dense calcifications, NIRS-IVUS could be valuable in predicting IVL’s impact on calcified lesions. Another consideration is that IVL was more effective in sites with lipid-rich calcifications, as these tended to have relatively thinner thickness compared to non-lipid calcified areas. However, the lipid calcifications in present case exceeded 500 µm, which is generally considered thick. Further investigation in this area is warranted.

Several reports have been described on the combination therapy of RA and IVL.^13,14^ Rotational atherectomy effectively improves device delivery by ablating the surface of the calcification and lumen gain, whereas IVL disrupts deep calcification. These two modalities have synergistic effects and may yield improved results for treating severely calcified lesions. Another significant benefit is that combining RA with small burrs and IVL could prevent serious procedural complications and femoral artery puncture in patients with reduced LVEF and high bleeding risk,^15^ as was demonstrated in this case. Even if conducted as separate procedures, the combination of RA and IVL for the same lesion could yield procedural and clinical outcomes, warranting exploration in forthcoming studies.

## Lead author biography


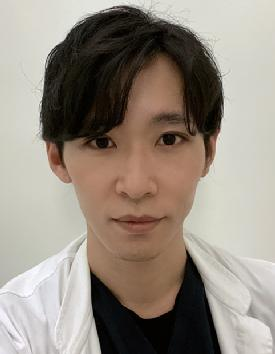


Dr Yusuke Miura, MD, PhD, graduated from Kyorin University in 2015. Now, he works as a cardiologist at the Department of Cardiovascular Medicine, Kyorin University Hospital. His research interest is percutaneous coronary intervention and intravascular imaging

## Supplementary Material

ytae504_Supplementary_Data

## Data Availability

The data that support the findings of this study are available from the corresponding author upon reasonable request.

## References

[1] Genereux P, Madhavan MV, Mintz GS, Maehara A, Palmerini T, Lasalle L, et al Ischemic outcomes after coronary intervention of calcified vessels in acute coronary syndromes. Pooled analysis from the HORIZONS-AMI (harmonizing outcomes with revascularization and stents in acute myocardial infarction) and ACUITY (acute catheterization and urgent intervention triage strategy) TRIALS. J Am Coll Cardiol 2014;63:1845–1854. 24561145 10.1016/j.jacc.2014.01.034

[2] Guedeney P, Claessen BE, Mehran R, Mintz GS, Liu M, Sorrentino S, et al Coronary calcification and long-term outcomes according to drug-eluting stent generation. JACC Cardiovasc Interv 2020;13:1417–1428. 32553329 10.1016/j.jcin.2020.03.053

[3] Brinton TJ, Ali ZA, Hill JM, Meredith IT, Maehara A, Illindala U, et al Feasibility of shockwave coronary intravascular lithotripsy for the treatment of calcified coronary stenoses. Circulation 2019;139:834–836. 30715944 10.1161/CIRCULATIONAHA.118.036531

[4] Bortnick AE, Epps KC, Selzer F, Anwaruddin S, Marroquin OC, Srinivas V, et al Five-year follow-up of patients treated for coronary artery disease in the face of an increasing burden of co-morbidity and disease complexity (from the NHLBI Dynamic Registry). Am J Cardiol 2014;113:573–579. 24388624 10.1016/j.amjcard.2013.10.039 PMC3946649

[5] Madhavan MV, Tarigopula M, Mintz GS, Maehara A, Stone GW, Genereux P. Coronary artery calcification: pathogenesis and prognostic implications. J Am Coll Cardiol 2014;63:1703–1714. 24530667 10.1016/j.jacc.2014.01.017

[6] Chambers JW, Feldman RL, Himmelstein SI, Bhatheja R, Villa AE, Strickman NE, et al Pivotal trial to evaluate the safety and efficacy of the orbital atherectomy system in treating de novo, severely calcified coronary lesions (ORBIT II). JACC Cardiovasc Interv 2014;7:510–518. 24852804 10.1016/j.jcin.2014.01.158

[7] de Waha S, Allali A, Buttner HJ, Toelg R, Geist V, Neumann FJ, et al Rotational atherectomy before paclitaxel-eluting stent implantation in complex calcified coronary lesions: two-year clinical outcome of the randomized ROTAXUS trial. Catheter Cardiovasc Interv 2016;87:691–700. 26525804 10.1002/ccd.26290

[8] Basavarajaiah S, Ielasi A, Raja W, Naneishvili T, Testa L, Popolo Rubbio A, et al Long-term outcomes following intravascular lithotripsy (IVL) for calcified coronary lesions: a real-world multicenter European study. Catheter Cardiovasc Interv 2023;101:250–260. 36525378 10.1002/ccd.30519

[9] Ali ZA, Nef H, Escaned J, Werner N, Banning AP, Hill JM, et al Safety and effectiveness of coronary intravascular lithotripsy for treatment of severely calcified coronary stenoses: the Disrupt CAD II Study. Circ Cardiovasc Interv 2019;12:e008434. 31553205 10.1161/CIRCINTERVENTIONS.119.008434

[10] Fujino A, Mintz GS, Matsumura M, Lee T, Kim SY, Hoshino M, et al A new optical coherence tomography-based calcium scoring system to predict stent underexpansion. EuroIntervention 2018;13:e2182–e2189. 29400655 10.4244/EIJ-D-17-00962

[11] Madder RD, VanOosterhout S, Klungle D, Mulder A, Elmore M, Decker JM, et al Multimodality intracoronary imaging with near-infrared spectroscopy and intravascular ultrasound in asymptomatic individuals with high calcium scores. Circ Cardiovasc Imaging 2017;10:e006282. 28982647 10.1161/CIRCIMAGING.117.006282

[12] Ijichi T, Nakazawa G, Torii S, Nakano M, Yoshikawa A, Morino Y, et al Evaluation of coronary arterial calcification—ex-vivo assessment by optical frequency domain imaging. Atherosclerosis 2015;243:242–247. 26408928 10.1016/j.atherosclerosis.2015.09.002

[13] Ielasi A, Loffi M, De Blasio G, Tespili M. Rota-Tripsy’: a successful combined approach for the treatment of a long and heavily calcified coronary lesion. Cardiovasc Revasc Med 2020;21:152–154. 31883981 10.1016/j.carrev.2019.12.023

[14] Buono A, Basavarajaiah S, Choudhury A, Lee L, Bhatia G, Hailan A, et al RotaTripsy’ for severe calcified coronary artery lesions: insights from a real-world multicenter cohort. Cardiovasc Revasc Med 2022;37:78–81. 34244087 10.1016/j.carrev.2021.06.132

[15] Urban P, Mehran R, Colleran R, Angiolillo DJ, Byrne RA, Capodanno D, et al Defining high bleeding risk in patients undergoing percutaneous coronary intervention: a consensus document from the Academic Research Consortium for High Bleeding Risk. Eur Heart J 2019;40:2632–2653. 31116395 10.1093/eurheartj/ehz372 PMC6736433

